# Supplemental Effects of Phytase on Modulation of Mucosa-Associated Microbiota in the Jejunum and the Impacts on Nutrient Digestibility, Intestinal Morphology, and Bone Parameters in Broiler Chickens

**DOI:** 10.3390/ani11123351

**Published:** 2021-11-24

**Authors:** Vitor Hugo C. Moita, Marcos Elias Duarte, Sung Woo Kim

**Affiliations:** Department of Animal Science, North Carolina State University, Raleigh, NC 27695, USA; vccardos@ncsu.edu (V.H.C.M.); mduarte@ncsu.edu (M.E.D.)

**Keywords:** apparent ileal digestibility, bone breaking strength, broiler chickens, intestinal health, mucosa-associated microbiota, phytase

## Abstract

**Simple Summary:**

A positive modulation of the gut microbiota is associated with benefits regarding intestinal health and subsequent growth performance. The supplementation of phytase has been studied for improving nutrient digestibility, bone parameters, and growth performance. This study aimed to determine the effects of increasing doses of phytase on the modulation of mucosa-associated microbiota in the jejunum enhancing intestinal health and the impacts on nutrient digestibility, bone parameters, and growth performance of broiler chickens. It was demonstrated that the use of phytase enhanced the intestinal health of the broiler chickens by potentially increasing beneficial and reducing harmful bacteria, enhancing intestinal morphology, and consequently improving nutrient digestibility and bone parameters. Our results suggest that the use of phytase can positively modulate the jejunal mucosa-associated microbiota in the jejunum, enhance intestinal integrity, nutrient digestibility, and bone parameters of broiler chickens.

**Abstract:**

This study aimed to determine supplemental effects of phytase on modulation of the mucosa-associated microbiota in the jejunum, intestinal morphology, nutrient digestibility, bone parameters, and growth performance of broiler chickens. Three hundred and sixty newly hatched broiler chickens (Ross 308) (44 ± 2 g BW) were randomly allotted in 6 treatments with 10 birds per cage based on a completely randomized design and fed for 27 d. The treatments consisted of one negative control (NC), diet formulated meeting the requirements suggested by Ross recommendations (2019), and without phytase supplementation. The other treatments consisted of a positive control diet (PC) formulated with 0.15% deficient Ca and P and split into 5 treatments with different phytase inclusion levels (0, 500, 1000, 2000, 4000 FTU/kg feed). Titanium dioxide (0.4%) was added to feeds as an indigestible marker to measure apparent ileal digestibility (AID) of nutrients. On d 27, 3 birds were randomly selected from each cage and euthanized to collect samples for analyzing the mucosa-associated microbiota in the jejunum, oxidative stress status, AID, and bone parameters. Data were analyzed using the proc Mixed of SAS 9.4. Phytase supplementation tended to have a quadratic effect (*p* = 0.078) on the overall ADG (maximum: 41 g/d at 2833 FTU/kg of feed). Supplementation of phytase at 2,000 FTU/kg increased (*p* < 0.05) the relative abundance of *Lactobacillus* and reduced (*p* < 0.05) *Pelomonas*. Moreover, it tended to reduce *Helicobacter* (*p* = 0.085), *Pseudomonas* (*p* = 0.090) *Sphingomonas* (*p* = 0.071). Phytase supplementation increased (*p* < 0.05) the villus height and the AID of CP; and tended to increase (*p* = 0.086) the AID of P. Phytase supplementation increased (*p* < 0.05) breaking strength and P content in the tibia. In conclusion, phytase supplementation showed potential benefits on the modulation of the mucosa-associated microbiota in the jejunum by tending to reduce harmful bacteria (*Pelomonas*, *Helicobacter,* and *Pseudomonas*) and increase beneficial bacteria (*Lactobacillus*). In addition, it showed positive effects increasing apparent ileal digestibility of CP and P, enhancing intestinal morphology (villus height), and improving the bone parameters (bone breaking strength, ash, and P content). Phytase supplementation at a range of 38 to 59 FTU/d or 600 to 950 FTU/kg of feed provided the most benefits related to nutrient digestibility.

## 1. Introduction

Enzymes have been widely used in animal production for optimizing nutrient digestibility and growth performance in all animal species. Exogenous enzymes supplemented with monogastric diets can contribute to the removal of antinutritional factors, modulating the intestinal microbiota, increasing the digestibility of nutrients and their utilization leading to improvements in intestinal health and growth performance [1,2,3].

Around 61–70% of the phosphorus (P) in cereal grains and oilseeds used in monogastric diet formulation are present in the form of phytic acid [4]. However, monogastric animals do not produce endogenous phytase and, consequently, the P content in the phytic acid form is not available for utilization by poultry [4,5]. Furthermore, phytic acid can also bind to proteins and enzymes like trypsin and α-amylase, inhibiting their activity and lowering protein and carbohydrate digestibility [6,7,8]. The undigested nutrients would be eliminated via excreta by the animals, raising an environmental concern [9]. When phytase is included in feeds there is a reduction in supplemental levels of calcium (Ca) and P to compensate for the increased uptake of these minerals provided by phytase supplementation [10,11]. A high supplementation of Ca alter the physical and chemical properties of the digesta in the gastrointestinal tract and lead to alterations in pH and solubility, which can affect the relative abundance and diversity of the intestinal microbiota [12,13].

The dietary inclusion of phytase can release the P in the phytic acid form, implying the degradation of insoluble antinutritional inositol hexaphosphoric acid and subsequent generation of lower inositol phosphates into inositol. [14,15,16]. In principle, phytase can hydrolyze phytate and convert it to lower myo-inositol phosphate esters, inositol, and inorganic P through a sequence of stepwise dephosphorylation reactions [16,17,18]. Part of the benefit of supplementing phytase could be attributed to catalyzing hydrolysis reaction to release inorganic P from phytate and consequently the generation of inositol from the hydrolysis of phytate [14,16,19].

The supplementation of phytase for poultry has been widely studied for different benefits, such as bone parameters, growth performance, nutrient digestibility, and intestinal health [14,20,21,22]. However, some studies reported that the supplementation of phytase can also play an important role in modulating the intestinal microbiota by reducing harmful and increasing beneficial bacteria, which in turn can exert benefits associated with nutrient digestibility, intestinal morphology, and bone parameters of broiler chickens [23,24,25]. The role of dietary phytase on the intestinal microbiota can be associated with the buffering property and availability of Ca and P for microbial fermentation [23,24,25]. According to Ptak et al. [23], the Ca and P released by hydrolysis of phytate by phytase increased the proliferation of lactic acid bacteria in broiler fed low Ca and P diet. The higher concentration of short-chain fatty acids and lactic acid can improve the intestinal environment, consequently, enhancing intestinal health and nutrient digestibility [23,25]. In addition, by hydrolyzing the phytate other minerals and nutrients that were complexed with the phytate molecule can be released further improving bone parameters and intestinal morphology [23,24,25].

Thus, it was hypothesized that the supplementation of phytase could positively modulate the mucosa-associated microbiota in the jejunum by increasing beneficial and reducing pathogen bacteria and consequently enhancing intestinal morphology nutrient digestibility and bone parameters in broiler chickens. Therefore, the objectives of this study were to determine the effects of increasing doses of phytase on the modulation of the mucosa-associated microbiota in the jejunum and the impacts on the nutrient digestibility, intestinal morphology, bone parameters, and growth performance of broiler chickens.

## 2. Materials and Methods

### 2.1. Animals, Design, and Diets

The experimental protocol was approved by the Institutional Animal Care and Use Committee of North Carolina State University. The experiment was conducted at the North Carolina State University Scott Hall (Raleigh, NC, USA). A total of 360 (half male and half female) newly hatched Ross 308 broiler chickens hatched from broiler breeders maintained at the Piedmont Research Station (Salisbury, NC, USA) were used in this study. On the first day of trial, birds were weighed and randomly allotted into cages containing 10 birds per cage based on a completely randomized design into 6 dietary treatments. The cage was considered the experimental unit. The 6 dietary treatments (Table 1) consisted of one negative control (NC), diet formulated meeting the requirements suggested by Ross recommendations (2019) and without phytase supplementation. The other treatments consisted of a positive control diet (PC) formulated with 0.15% deficient Ca and P and split into 5 treatments with different phytase inclusion levels (0, 500, 1000, 2000, 4000 FTU/kg feed). Before mixing to the experimental diets, the phytase (VTR BIOTECH CO., LTD, Guangdong, China) was premixed with ground corn. One FTU is defined as the activity that releases 1 μmol of inorganic phosphate from 5.0 mM sodium phytate per minute at pH 5.5 and 37 °C. The birds had ad libitum access to water and feed throughout the study. Titanium dioxide (0.4%) was added to the diets as an indigestible external marker to determine the apparent ileal digestibility (AID) of nutrients.

### 2.2. Growth Performance

The BW and feed intake were recorded at the end of each week to calculate the average BW, ADG, ADFI, and G:F as indicators of growth performance.

### 2.3. Sample Collection and Processing

After 27 d feeding, 3 birds were randomly selected from each cage to be euthanized by CO_2_ asphyxiation to collect jejunal mucosa to measure oxidative stress parameters, and diversity and relative abundance of the mucosa-associated microbiota; jejunal tissues to measure intestinal morphology; ileal digesta to measure AID of nutrients; and the left tibia to measure bone breaking strength and mineral content. Mucosal samples from mid-jejunum were scraped, pooled, and placed into 2 mL tubes, and later stored at −80 °C (after snap-freezing in liquid nitrogen, immediately after collection). For measuring the AID of dry matter (DM), crude protein (CP, 6.25 × N), Ca, and P ileal digesta was collected in the region from the posterior 2/3 of the Meckel’s diverticulum to about 2 cm anterior to the ileocecal junction. The ileal digesta was collected and pooled by gently squeezing and stored into 100 mL containers and placed on ice, and then stored at −20 °C for further analysis. A section of approximately 5 cm from the mid-jejunum was taken, flushed with a 0.9% saline solution, and placed in 50 mL tubes with 40 mL of 10% formalin to be fixed for further microscopic assessment of intestinal morphology. The left tibia bone was collected from the same birds. Fat was extracted from the tibias using pure petroleum ether and subsequently ashed to be analyzed for P by spectrophotometry and Ca by flame atomic absorption spectroscopy as previously described by Babatunde et al. [21].

### 2.4. Diversity and Relative Abundance of the Mucosa-Associated Microbiota in the Jejunum

Mucosa samples collected from the mid-jejunum were used for microbiome sequencing using the 16S rRNA gene sequence analysis. The DNA was extracted using the DNA Stool Mini Kit (# 51604, Qiagen; Germantown, MD, USA) following the instructions of the manufacturer. The extracted DNA samples were sent to Mako Medical Laboratories (Raleigh, NC, USA) for 16S rRNA gene sequencing. The extracted DNA samples were prepared for the template on the Ion Chef Instrument and sequencing on the Ion S5TM system (ThermoFisher Scientific, Waltham, MA, USA). Variable regions V2, V3, V4, V6, V7, V8, and V9 of the 16S rRNA gene were amplified using Ion 16S Metagenomics Kit (ThermoFisher Scientific, Waltham, MA, USA). Sequences (Hypervariable regions) were processed via Torrent Suite Software (version 5.2.2) (ThermoFisher Scientific, Waltham, MA, USA) to produce bam files for further analysis. Sequence data analysis, alignment to GreenGenes and MicroSeq databases, alpha and beta diversity plot generation, and OTU table generation were performed by the Ion Reporter Software Suite (version 5.2.2) of bioinformatics analysis tools (ThermoFisher Scientific, Waltham, MA, USA). The Ion Reporter’s Metagenomics 16S workflow powered by Qiime (version w1.1) was used to analyze the samples. To initiate the statistical analysis of the microbiota, OTU data were transformed to relative abundance as previously described by Duarte et al. [26]. The OTU with the relative abundance < 0.5% within each level were combined as “Others”.

### 2.5. Oxidative Stress Parameters

Malondialdehyde was measured following OxiSelect TBARS MDA Quantitation Assay Kit (#STA-330, Cell Biolabs, Inc., San Diego, CA, USA). The concentration range of MDA standards was 0 to 125 μM. The absorbance was measured at 540 nm. The concentration of MDA was calculated based on the standard curve created from the concentration and absorbance of the respective standard and described as μmol/g of protein, as described by Zhao et al. [27].

Protein carbonyl was measured following OxiSelect Protein Carbonyl ELISA Kit (#STA-310, Cell Biolabs, Inc., San Diego, CA, USA). All samples were diluted using PBS to reach the protein concentration of 10 μg/mL. The working range of standards was 0.375 to 7.5 nmol/mg protein. The absorbance was measured at 540 nm. The concentration of protein carbonyl was calculated based on the standard curve created from the concentration and absorbance of the respective standard and described as nmol/mg of protein, following Zhao et al. [28].

### 2.6. Apparent Ileal Digestibility

The frozen ileal digesta samples were dried using a freeze dryer machine (24D × 48, Virtis, Gardiner, NY, USA). Dried digesta and feed samples were ground to powder form and stored in plastic containers at −20 °C for further analysis. Titanium dioxide concentration in the feed and digesta was measured as previously described by Moita et al. [21]. The working range of the standards was 0 to 10 mg of titanium dioxide. Samples were weighed around 0.5 g onto a tarred weighing paper and then placed into 75 mL digestion tubes. One Kjeltab tablet (ThermoFisher Scientific, Waltham, MA, USA) and five pieces of selenized boiling granules were added to each digestion tube to prevent explosive vaporization. After adding 10 mL of concentrated H_2_SO_4_ (sulfuric acid), all digestion tubes were vortexed immediately. Then the tubes were heated for 2.5 h at 420 °C under a fume hood. When tubes got cool after 30 minutes at room temperature, 2 mL of 30% H_2_O_2_ (hydrogen peroxide) was added to each tube four times and were vortexed until yellow to orange color appeared. Deionized water was added until the volumetric mark was reached and then the tubes were covered and gently mixed. After that, 200 µL from the tubes were pipetted to a 96 well plate, which was read immediately at 410 nm. Titanium dioxide values were calculated based on the standard curve created from the concentration and absorbance of the respective standards. The feed and digesta samples were weighed around 0.5 g to analyze the nitrogen content using TruSpec N Nitrogen Determinator (LECO CN-2000, LECO Corp., St. Joseph, MI, USA) to later obtain the CP (6.25 × N). Feed and digesta samples were also analyzed for determining DM (Method 934.01, AOAC, 2006), P by spectrophotometry (AOAC Official Method 946.06) and Ca by flame atomic absorption spectroscopy (AOAC Official Method 968.08). Apparent ileal digestibility of DM, CP, Ca, and P were calculated using the following equation previously described by Chen et al. [22]:

AID (%) = {1 − [(TiO_2_feed/TiO_2_digesta) × (Nutrientdigesta/Nutrientfeed)]} × 100

where TiO_2_feed represents the titanium dioxide concentration in the feed, TiO_2_digesta is the titanium dioxide concentration in the ileal digesta, Nutrientfeed represents the nutrient concentration in the feed, and Nutrientdigesta is the nutrient concentration in the ileal digesta.

### 2.7. Intestinal Morphology

After being fixed in 10% formalin for 48 h, two portions of 5-mM thick slides were taken from the jejunum section and placed in cassettes that were reserved with 70% of ethanol solution. The samples were sent to the North Carolina State University Histology Laboratory (Raleigh, NC, USA). Then, the samples were dehydrated, embedded in paraffin, cut cross-section to 5 µm thick, and mounted on polylysine-coated slides. Villus height, villus width, and crypt depth were measured using a microscope Olympus CX31 (Lumenera Corporation, Ottawa, ON, Canada) with a camera Infinity 2–2 digital CCD. Lengths of 10 well-oriented intact villi and their associated crypts were measured in each slide. The villus length was measured from the top of the villus to the villus-crypt junction, the villus width was measured in the middle of the villus, and crypt depth was measured from the villus-crypt junction to the bottom of the crypt. Moreover, the villus height and crypt depth ratio (VH:CD) were calculated. The averages of the 10 measurements per cage were used for the statistical analysis considering the cage as the experimental unit. All the analyses of the intestinal morphology were executed by the same person, as previously described by Shen et al. [20].

### 2.8. Bone Parameters

The left tibia was separated and removed of all adhering soft tissue and cartilaginous end caps to measure bone breaking strength and composition, and ash weights. The bone-breaking strength of the tibias was analyzed right after collection using and it was tested by using an axial servo-hydraulic load frame (858 Mini Bionix II, MTS Systems Inc., Minneapolis, MN, USA). The instrument measures newton (N) of force required to break tibias placed on 2 supports spaced 2.0 cm apart when force was applied to the center of the bone by an instrument moving at 30 mm/min. The breaking strength was measured by a pressure-sensitive cell and recorded on a graph recorder in newton’s (N). After bone breaking strength assay the fat was removed from the tibias using pure petroleum ether following AOAC Official Method 932.16 [29]. Fat-extracted tibias were then dried for 24 h and subsequently ashed at 600 °C for 24 h. Ashed samples were analyzed for the concentration of P by spectrophotometry AOAC Official Method 946.06 [29] and Ca by flame atomic absorption spectroscopy AOAC Official Method 968.08 [29].

### 2.9. Statistical Analysis

Data were analyzed based on a completely randomized design using the MIXED procedure of the SAS 9.4 software (SAS Inc., Cary, NC, USA). The cage was considered the experimental unit as birds were fed together with a single feeder in cages. Linear and quadratic effects of phytase supplementation were tested by polynomial contrasts. Coefficients for unequally spaced concentrations of supplemental phytase were obtained using the IML procedure. For the growth performance, AID of nutrients, intestinal morphology, oxidative stress status, and bone parameters data, pre-planned contrasts were established to compare supplementation ranges of phytase, the positive and negative control treatments (NC vs. 0, 0 vs. Phy, 0 vs. 1000 to 4000 FTU/kg feed and 0 vs. 2000 to 4000 FTU/kg feed). When significant or tendency effects were found among contrasts, the data were further analyzed using the NLMIXED procedure to determine the breaking point for obtaining the optimal phytase supplemental level, as previously described by Robbins et al. [30] and Jang et al. [31]. The NC treatment was not included in the broken-line analysis. The predictor was set by multiplying the phytase inclusion (FTU/kg feed) with the ADFI (0.062 kg/d) to account for the feed consumption of the animals through the experimental period. After the breakpoint was found, it was converted back from FTU/d to FTU/kg feed by dividing with the ADFI (0.062 kg/d). For the broken-line model, the *p*-value of each parameter indicates if the changes in the parameters are associated with the changes in the response. For the data analysis of relative abundance and diversity of mucosa-associated microbiota, contrasts were established to compare the supplementation of phytase (NC vs. 0, 0 vs. 2000, NC vs. 2000), based on Lee et al. [32,33]. Statistical differences were considered significant with *p* < 0.05 and tendency with 0.05 ≤ *p* < 0.10.

## 3. Results

### 3.1. Growth Performance

The growth performance was not affected when the NC and the PC treatments were compared during the overall experimental period (Table 2). However, increasing supplementation of phytase tended to have a quadratic effect on the ADG on the periods from d 7 to d 27 (maximum: 51 g/d at 2625 FTU/kg of feed) (*p* = 0.058) and from d 1 to d 27 (maximum: 41 g/d at 2833 FTU/kg of feed) (*p* = 0.078). The ADFI and G:F were not affected by the supplementation of phytase.

### 3.2. Diversity and Relative Abundance of the Mucosa-Associated Microbiota in the Jejunum

Phytase supplementation at 2000 FTU/kg feed increased (*p* < 0.05) and tended to increase (*p* = 0.091) the alpha diversity of the mucosa-associated microbiota in jejunum at family and genus level estimated with the Chao1 index, respectively when compared with the PC treatment (Table 3). No differences were observed between treatments on the other alpha-diversity indexes.

At the phylum level (Table 4), no differences in the relative abundance of the mucosa-associated microbiota in the jejunum were observed between the treatments.

At the family level (Table 5), it was observed a tendency for PC treatment to increase the relative abundance of *Helicobacteraceae* (*p* = 0.054) when compared with NC treatment. Furthermore, it was observed that phytase supplementation at 2000 FTU/kg of feed tended to reduce the relative abundance of *Propionibacteriaceae* (*p* = 0.078) and *Helicobacteraceae* (*p* = 0.055) when compared with the PC treatment. Phytase supplementation at 2000 FTU/kg feed tended to decrease (*p* = 0.095) the relative abundance of *Enterobacteriaceae* when compared with the NC treatment.

At the genus level (Table 6), it was observed a tendency for PC to increase the relative abundance of *Staphylococcus* (*p* = 0.084) and *Helicobacter* (*p* = 0.085) when compared with NC treatment. Phytase supplementation at 2000 FTU/kg feed tended to increase (*p* = 0.099) the relative abundance of *Lactobacillus* when compared with the PC treatment. Moreover, phytase supplementation at 2000 FTU/kg feed decreased (*p* < 0.05) the relative abundance of *Pelomonas* and tended to decrease the relative abundance of *Microbacterium* (*p* = 0.080) and *Methylobacterium* (*p* = 0.065) when compared with the PC treatment. In the same way, phytase supplementation at 2,000 FTU/kg feed tended to reduce the relative abundance of *Pseudomonas* (*p* = 0.090), *Methylobacterium* (*p* = 0.092), and *Sphingomonas* (*p* = 0.071) when compared with the NC treatment.

At the species level (Table 7), phytase supplementation at 2000 FTU/kg of feed decreased (*p* < 0.05) the relative abundance of *Microbacterium_ginsengisoli* when compared with the NC treatment and tended to decrease (*p* = 0.068) the relative abundance of the same specie when compared with PC treatment. Phytase supplementation at 2000 FTU/kg feed increased (*p* < 0.05) the relative abundance of *Pelomonas_puraquae* and tended to increase the relative abundance of *Lactobacillus_vaginalis* (*p* = 0.054), *Lactobacillus_reuteri* (*p* = 0.097), and *Acinetobacter_johnsonii* (*p* = 0.084) when compared with the PC treatment. Moreover, PC treatment decreased (*p* < 0.05) the relative abundance of *Lactobacillus_vaginalis* and tended to decrease (*p* = 0.083) the relative abundance of *Lactobacillus_reuteri*. Furthermore, PC treatment tended to reduce the relative abundance of *Helicobacter_mastomyrinus* (*p* = 0.094) and *Clostridium_perfringens* (*p* = 0.065).

### 3.3. Oxidative Stress Parameters

The oxidative stress parameters of broiler chickens were not affected by the phytase supplementation (Table 8).

### 3.4. Apparent Ileal Digestibility

**Figure 1:**
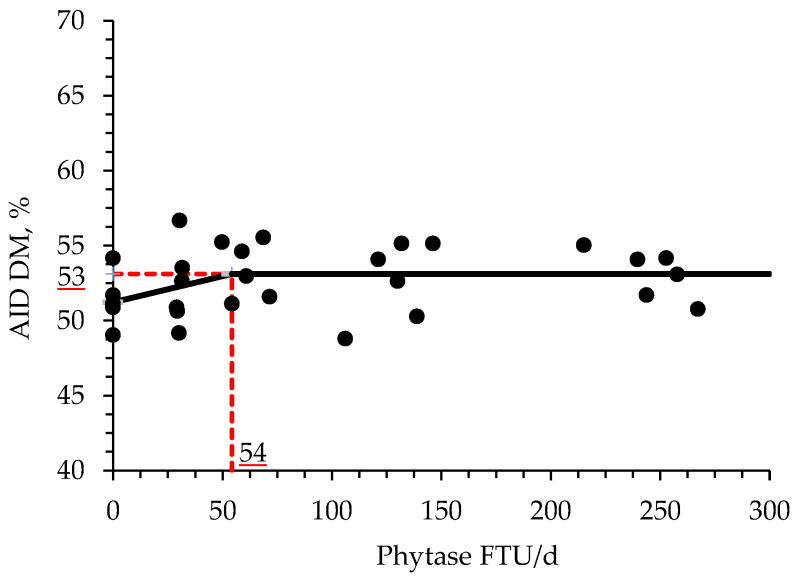
Changes in the AID of DM with supplementation of phytase using a broken-line analysis. The NC treatment was not included in this model. The breakpoint was 54 FTU/d of feed of phytase supplementation when the bone-breaking strength was 53%. The equation for AID of DM was Y = 53−0.03488 × zl; if phytase supplementation is ≥breakpoint, then z; = 0; if phytase supplementation is <breakpoint, then zl = breakpoint—phytase intake. Values for phytase activity were based on the analyzed values. *p*-value for the intercept was <0.05, for the slope was <0.05 and for the breaking point was <0.05 (confidence interval 95%: 54.01 to 54.55; SE: 0.13). The breakpoint was converted from 54 FTU/d to 871 FTU/kg feed by dividing with the overall average feed intake (0.062 kg/d).

**Figure 2:**
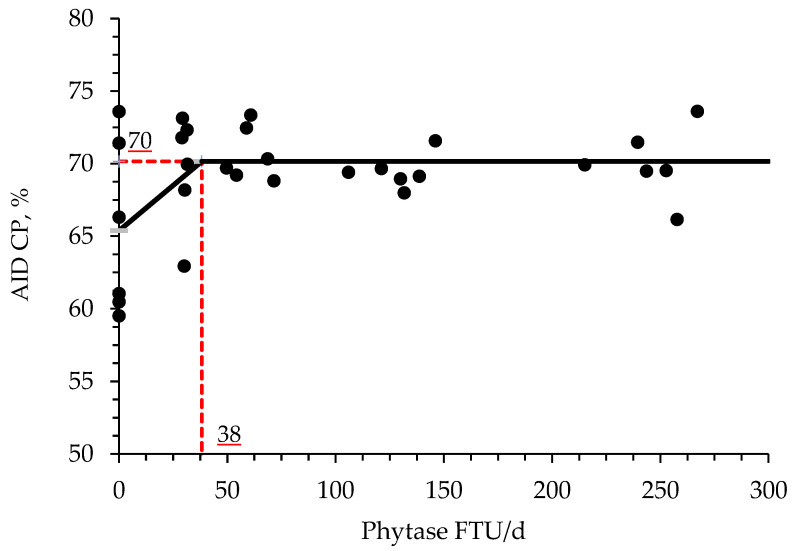
Changes in the AID of CP with supplementation of phytase using a broken-line analysis. The NC treatment was not included in this model. The breakpoint was 38 FTU/d of phytase supplementation when the AID of CP was 70%. The equation for AID of CP was Y = 70−0.1249 × zl; if phytase supplementation is ≥breakpoint, then z; = 0; if phytase supplementation is <breakpoint, then zl = breakpoint—phytase intake. Values for phytase activity were based on the analyzed values. The *p*-value for the intercept was <0.05, for the slope was 0.058 and for the breaking point was 0.021 (confidence interval 95%: 6.10 to 70.31; SE: 15.72). The breakpoint was converted from 54 FTU/d to 871 FTU/kg feed by dividing with the overall average feed intake (0.062 kg/d).

**Figure 3:**
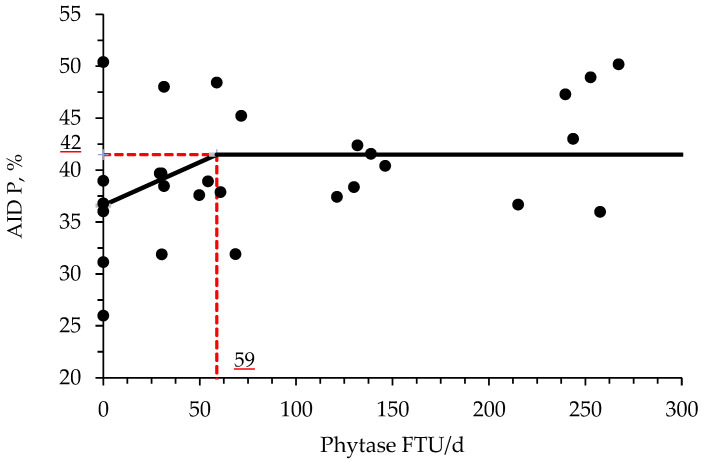
Changes in the AID of P with supplementation of phytase using a broken-line analysis. The NC treatment was not included in this model. The breakpoint was 59 FTU/d of phytase supplementation when AID of P was 42%. The equation for AID of P was Y = 42−0.08318 × zl; if phytase supplementation is ≥breakpoint, then z; = 0; if phytase supplementation is <breakpoint, then zl = breakpoint—phytase intake. Values for phytase activity were based on the analyzed values. The *p*-value for the intercept was <0.05, for the slope was 0.077 and for the breaking point was <0.05 (confidence interval 95%: 58.55 to 60.59; SE: 0.14). The breakpoint was converted from 59 FTU/d to 952 FTU/kg feed by dividing with the overall average feed intake (0.062 kg/d).

The supplementation of increasing levels of phytase increased (*p* < 0.05) the AID of CP and P when compared with PC treatment (Table 9). Moreover, the birds fed with NC increased (*p* < 0.05) the AID of DM and tended to increase (*p* = 0.092) the AID of P when compared with PC treatment. Phytase supplementation at a range from 1000 to 4000 and from 2000 to 4000 FTU/kg feed increased (*p* < 0.05) the AID of CP when compared with the PC treatment. Phytase supplementation at a range from 1000 to 4000 tended to increase the AID of P (*p* = 0.086) and DM (*p* = 0.088) when compared with PC treatment. Furthermore, phytase supplementation at a range from 2000 to 4000 FTU/kg feed increased (*p* < 0.05) the AID of CP and tended (*p* = 0.070) to increase the AID of P when compared with PC treatment. The broken line analysis on the AID of DM of broiler chickens with different phytase supplemental levels indicated that the optimal phytase supplemental level is 54 FTU/d or 872 FTU/kg of feed (Figure 1). The broken line analysis on the AID of CP of broiler chickens with different phytase supplemental levels indicated that the optimal phytase supplemental level is 38 FTU/d or 614 FTU/kg of feed (Figure 2). The broken line analysis on the AID of P of broiler chickens with different phytase supplemental levels indicated that the optimal phytase supplemental level is 59 FTU/d or 952 FTU/kg of feed (Figure 3).

### 3.5. Intestinal Morphology

The supplementation of increasing levels of phytase increased (*p* < 0.05) the villus height, and villus width of the broiler chickens (Table 10). Moreover, the supplementation of increasing levels of phytase had a quadratic effect (*p* < 0.05) on the crypt depth (minimum: 167 µm at 1900 FTU/kg of feed). Phytase supplementation at a range from 1000 to 4000 and from 2000 to 4000 FTU/kg feed increased (*p* < 0.05) the villus height and villus width when compared with the PC treatment. On the other hand, phytase supplementation at a range from 2000 to 4000 FTU/kg feed tended (*p* = 0.071) to increase the crypt depth when compared with PC treatment.

### 3.6. Bone Parameters

The bone-breaking strength of birds fed with NC was higher (*p* < 0.05) than birds fed with PC (Table 11). Whereas the bone-breaking strength of broiler chickens supplemented with phytase tended to linearly increase (*p* = 0.073) and to have a quadratic effect (*p* = 0.096) (maximum: 217.3 N at 2713 FTU/kg of feed). The % of ashes of the animals fed with NC tended (*p* = 0.093) to be higher when compared with animals fed with PC. Moreover, the tibia P content was higher (*p* < 0.05) on animals fed with NC when compared with animals fed with PC. Phytase supplementation at a range from 1000 to 4000 and from 2000 to 4000 FTU/kg feed increased (*p* < 0.05) the bone-breaking strength, ash per gram of tibia, ash, and P content when compared with the PC treatment.

## 4. Discussion

Phytase supplementation for broiler diets has been associated with improvements in growth performance, nutrient digestibility, bone parameters, and its efficacy has been established with a large number of studies over time [8,22,34,35]. Recently, the study of the mucosa-associated microbiota in the jejunum has gained more attention due to the relevance for the metabolism of nutrients, stimulation of immune response, protection from pathogens, and stimulation of epithelium cell proliferation of the animals [25,36]. Modulation of the microbiota toward a more healthy one may reflect improvements in the health and productive performance of monogastric animals [3,23,24,37]. The microbiota composition can vary among species and sections of the organism, for example, the chicken’s intestinal microbiota is composed of more than 900 species [24,38].

In the present study, the mucosa-associated microbiota in the jejunum was mainly composed of four phyla with *Firmicutes*, *Proteobacteria*, *Cyanobacteria,* and *Actinobacteria* accounting for more than 85% of the total mucosa-associated microbiota in the jejunum. The intestinal microbiota can play an essential role in nutrient digestibility, hydrolysis of antinutritional factors and toxins, decrease in pathogens, stimulation, and modulation of the immune system and metabolism [24,39]. The solubility and stability of phytate-mineral complexes are both pH dependents [12,40,41], and most of these complexes are soluble at a low pH lower than 4 and insoluble between a pH 4 and 7 [12]. The formation of phytate-mineral complexes can impact phytase efficacy [12,42]. A low solubility may have an impact on phytate P digestibility and availability because is directly influenced by the hydrolysis of the phytate-mineral complexes [12,41] and because the pH of the broilers’ small intestinal is between 5.5 and 6.6, it can favor the formation of these complexes [12,13].

Mineral cations such as Zn^2+^, Fe^2+^, Mn^2+^, Fe^3+^, Ca^2+^, and Mg^2+^ have different properties regarding the formation and hydrolysis of phytate-mineral complexes [40]. According to Maenz et al. [40], zinc and iron have been reported with higher affinity and Ca and magnesium with lower affinity with the phytate molecule. Even though Ca ions have a low affinity with the phytate molecule, it may cause more impact on the hydrolysis of the phytate molecule because traditionally in poultry diets, Ca is a mineral added in a high concentration. Different authors attributed the negative effects of a high Ca supplementation on the hydrolysis of the phytate molecule due to a combination of factors [12,22]. It may be due to the precipitation of phytate by Ca through Ca-phytate-complex formation [12,40] or by an increase in the intestinal pH caused by the supplementation of Ca which will lead to a reduction of the mineral solubility and consequently availability [12,43]. A high supplementation of Ca can alter the physical and chemical properties of the digesta in the gastrointestinal tract [12,22]. Shafey et al. [43], found that by increasing Ca the pH of the digesta in the crop and ileum was increased, although alterations on the digesta pH may reflect on different shifts in the microbiota population.

A lower pH in the small intestine may provide a bacteriostatic effect, which is positive for intestinal integrity and the characteristics of the microbiota population by reducing the occurrence of pathogenic bacteria such as *Enterobacteriaceae* and *Heliboacteraceae*, and increasing beneficial bacteria, such as lactic acid bacteria populations [23,44]. These findings are in agreement with the results of the present study, where phytase supplementation tended to reduce the relative abundance of *Enterobacteriaceae* and *Heliboacteraceae* and to increase the relative abundance of *Lactobacillus*. Also, it tended to reduce the relative abundance of *Pelomonas*, *Microbacterium*, *Pseudomonas*, *Methylobacterium*, *Sphingomonas*. Furthermore, phytase supplementation tended to increase the relative abundance of *Lactobacillus reuteri*, which is a Gram-positive bacteria also known as lactic acid bacteria that can play a role as a probiotic for different species [45,46]. Different associated benefits were reported with a high abundance of this bacteria, such as the production of antimicrobial components (organic acids, ethanol, and reuterin) that can lead to inhibition of the colonization of pathogenic microbes and remodeling the commensal microbiota composition [45,47,48]. Additionally, it can strengthen the intestinal barrier and may decrease the microbial translocation from the gut lumen to the tissues [45,49]. However, it is important to keep in mind that different diet compositions can affect and interact with different microbial communities throughout the different parts of the digestive tract of the birds, which can primarily account for a large variation among the replicates [24].

A low microbial diversity can be associated with diets containing high levels of Ca and may lead to a decrease in the growth performance [24] and the energy value of the diet [50]. The bacterial growth can be affected by different Ca levels in a wide range of factors such as, by reducing acidic fermentation and precipitation of cytotoxic components in the intestinal lumen [23,51], by increasing gastric acid secretion [23,52], and by reducing pathogenic adhesion on the intestinal mucosal walls [23,53,54]. In the present study where we found that phytase supplementation increased the alpha diversity of the mucosa-associated microbiota in the jejunum at the family and tended to increase at the genus level estimated with the Chao1 index. Therefore, it may correlate the microbiota results and a positive effect of the phytase supplementation observed on the overall ADG of the birds. Furthermore, the insoluble complexes formed with Ca with phytate [24,55] will interact in the intestinal lumen with inorganic P resulting in the formation of Ca-orthophosphate [24,56], which can lead to a decrease in the productive performance of the birds due to the decreased solubility and availability of the P [24,57]. The positive effects of phytase supplementation for broiler chickens are well established and are considered as an alternative for reducing the use of inorganic P, reducing P excretion in the environment, improving nutrient digestibility and utilization, and the growth performance [20,21,58,59].

The primary established role of phytase was to increase the bioavailability and utilization of phytate-bound P, nevertheless, it may also provide new insights into the anti-nutritive properties of phytate. The intestinal jejunal morphology parameters, such as villus height (VH), villus width (VW), crypt depth (CD), and villus height crypt depth ratio (VH:CD), can be interpreted as indicatives of intestinal health in broiler chickens [60] since the intestinal jejunum is considered the main site of absorption in the small intestine [61]. According to Nari et al. [60], an increase in the villus height and VH:CD are linked to an enhanced epithelial cells turnover, and improvements on these parameters are correlated with activation of cell mitosis [62] and greater nutrient absorption and utilization. Birds with longer villi may be an indicator of a greater absorptive capacity and mature enterocytes in the lumen [63,64], whereas birds with higher crypt depth may represent a high cell turnover, which can be considered a response to epithelial destruction and inflammation [63,65]. In the present study, phytase supplementation increased the VH and VW of broiler chickens, which are in agreement with other studies that reported positive effects of phytase on the intestinal morphology parameters [60,66]. The increased VH and CD may also be related to the positive modulation of the mucosa-associated microbiota observed in the present study. On the other hand, the intestinal morphology data of crypt depth indicated that a higher supplementation might have deleterious effects on the intestinal morphology, however, it might be indirectly correlated with the health status and wellbeing of the birds, as previously described by Paiva et al. [63]. Supplementing phytase exogenously will express their positive effects regardless of the bird’s health status, although when a bird is not healthy their ability to digest and absorb nutrients will be compromised and consequently nutrients released by phytase will available for pathogenic bacteria leading to issues on the microbial population of the small intestine [63,67,68]. The intestinal morphology results of the present study may be an indicator of greater capacity of digestion, absorption, and utilization of nutrients by the birds supplemented with increasing levels of phytase.

Phytase supplementation is also involved with protein and amino acid digestion and availability for the animal due to the formation of protein-phytate complexes. The pH can be determinant for the formation of those complexes, which was first reported by Breese Jones and Csonka [69] and had that adverse effects for monogastric animals studied by Rojas and Scott [70]. According to Selle et al. [71], binary protein phytate-complexes are present at acidic pH, and ternary protein mineral phytate-complexes are formed via a cationic bridge as pH approaches neutrality. In theory, the hydrolysis of phytate will release phytate-bound proteins and P for animal utilization [41,71]. Phytase is capable of increasing the utilization of dietary amino acids and N by decreasing these antinutritional effects of the phytate molecule [41]. In addition, a reduction in the dietary Ca may improve the protein and amino acid digestibility and utilization by facilitating a decrease in gastric pH and consequently improving the pepsin efficiency [23,72]. The results of the present study are in agreement with these findings, where the AID of CP and P increased, whereas numerically increased the AID of Ca in broiler chickens supplemented with increasing levels of phytase. Moreover, it was found by broken-line analysis that the optimal supplemental levels of phytase for improving AID of CP, DM, and P are 33, 54, and 59 FTU/d or 541, 885, and 952 FTU/kg feed, respectively. During the preparation of enzymes, usually, the microorganisms used are genetically modified generating enzymatic side activities by the presence of other enzymes, such as proteases, in their structure [41]. Based on Selle et al. [71] findings, any enzymatic side activities present in the phytase structure are secondary, nevertheless, they may increase the hydrolysis of the phytate molecule, either by their own phosphoric effects or by improving the accessibility of phytase to its substrate.

Furthermore, phytase supplementation also shows potential benefits regarding bone parameters, such as the breaking strength, minerals, and ash content for monogastric animals, especially broiler chickens [14,22,57,59,73,74]. According to Sebastian et al. [75], the improvement of the ash percentage in the tibia may indicate an increase in bone mineralization consequent to an increase in mineral availability released by phytase from the phytate-mineral complexes. The results of the present study showed that the bone-breaking strength, ash, and P content of the bone increased, whereas the Ca content numerically increase in broiler chickens supplemented with increasing levels of phytase. Increasing the mineral content of the bone, especially P content, may reflect on a greater breaking strength indicating that phytase plays an important role in bone mineralization. These positive effects can be attributed to the efficacy of phytase in hydrolyzing the phytate molecule and consequently releasing more P and other nutrients bound to the phytate complexes.

Supplemental phytase can provide benefits that go beyond improving nutrient digestibility, bone parameters, and growth performance. In this study, the supplementation of increasing levels of phytase played an important role in the intestinal health of broiler chickens by potentially reducing pathogenic and increasing beneficial bacteria, which in turn may reflect in benefits on nutrient digestibility, intestinal morphology, and bone parameters. The supplementation of phytase may be an interesting alternative for improving intestinal health and subsequent performance of broiler chickens.

## 5. Conclusions

In conclusion, phytase supplementation showed potential benefits on the modulation of the mucosa-associated microbiota in the jejunum by tending to reduce harmful bacteria (*Pelomonas*, *Helicobacter,* and *Pseudomonas*) and increase beneficial bacteria (*Lactobacillus*). In addition, it showed positive effects increasing apparent ileal digestibility of CP and P, enhancing intestinal morphology by increasing villus height and width, and improving the bone parameters (bone breaking strength, ash, and P content). Phytase supplementation at a range of 38 to 59 FTU/d or 600 to 950 FTU/kg of feed provided the most benefits related to nutrient digestibility.

## Figures and Tables

**Table 1:** Composition of basal diets (as-fed basis).

Item	Treatment ^1^	
	NC	PC
Ingredients, %		
Corn, yellow dent	53.91	55.05
Soybean meal, 48% CP	34.00	34.00
Corn DDGS ^2^	4.00	4.00
Poultry meal	2.00	2.00
Dicalcium phosphate	2.00	1.02
Poultry fat	1.40	1.00
Limestone, ground	0.77	1.03
Titanium dioxide	0.40	0.40
DL-Met	0.32	0.31
Salt	0.30	0.30
L-Lys HCl	0.24	0.24
Choline, 60%	0.20	0.20
Mineral premix ^3^	0.15	0.15
Selenium premix	0.10	0.10
L-Thr	0.08	0.07
Phytase premix ^4^	0.08	0.08
Vitamin premix ^5^	0.05	0.05
Calculated composition		
ME ^6^, kcal/kg	3000	3000
CP ^7^, %	23.00	23.00
Digestible Lys, %	1.28	1.28
Digestible Met + Cys, %	0.95	0.95
Ca ^8^, %	0.96	0.81
*p* ^9^ available, %	0.48	0.33
*p* total, %	0.82	0.65
Analyzed composition		
DM ^10^, %	88.4 ± 0.4	88.4 ± 0.4
CP, %	23.2 ± 0.3	23.3 ± 0.4
Ca, %	1.08 ± 0.02	0.90 ± 0.10
Phytate ^11^, %	0.32 ± 0.01	0.37 ± 0.02
Total *p*, %	0.86 ± 0.02	0.67 ± 0.01

^1^ NC: no phytase, diet meeting nutrient requirements by Ross Nutrient Specification (2019); PC: no phytase, diet with Ca 0.15% and P with 0.15% lower than Ross Nutrient Specification (2019) requirements; ^2^ DDGS: corn distillers dried grains with soluble; ^3^ The trace mineral premix provided per kilogram of complete diet: 33 mg of Mn as manganous oxide, 110 mg of Fe as ferrous sulfate, 110 mg of Zn as zinc sulfate, 16.5 mg of Cu as copper sulfate, 0.30 mg of I as ethylenediamine dihydroiodide, and 0.30 mg of Se, as sodium selenite; ^4^ Phytase enzyme mixed with corn; ^5^ The vitamin premix provided per kilogram of complete diet: 6614 IU of vitamin A as vitamin A acetate, 992 IU of vitamin D3, 19.8 IU of vitamin E, 2.64 mg of vitamin K as menadione sodium bisulfate, 0.03 mg of vitamin B12, 4.63 mg of riboflavin, 18.52 mg of D-pantothenic acid as calcium panthonate, 24.96 mg of niacin, and 0.07 mg of biotin; ^6^ Metabolizable energy; ^7^ Crude protein; ^8^ Calcium; ^9^ Phosphorus; ^10^ Dry matter; ^11^ Phytate analysis was performed using the Megazyme kit K-PHYT 05/19 (Megazyme, Bray Business Park, Bray, Co. Wicklow, A98 YV29, Ireland).

**Table 2:** Growth performance of broiler chickens fed diets with phytase supplementation.

Item		Phytase, FTU/kg Feed						*p*-Value				
	NC ^1^	0 ^2^	500	1000	2000	4000	SEM	Linear	Quadratic	NC vs. 0	0 vs. 1000 to 4000	0 vs. 2000 to 4000
BW ^3^, g												
d 1	44	44	44	44	44	44	2	0.639	0.943	0.731	0.863	0.842
d 7	128	126	128	131	129	126	4	0.693	0.695	0.720	0.583	0.782
d 14	325	329	339	321	331	332	8	0.964	0.925	0.710	0.866	0.853
d 21	700	689	703	678	712	714	16	0.572	0.258	0.622	0.507	0.226
d 27	1155	1121	1111	1095	1167	1128	36	0.615	0.116	0.514	0.827	0.554
ADG ^4^, g/d												
d 1 to d 7	12.1	11.5	11.6	12.6	12.1	11.6	0.6	0.437	0.635	0.539	0.318	0.554
d 7 to d 14	28.2	29.9	30.1	27.2	30.0	29.4	1.0	0.665	0.415	0.216	0.354	0.861
d 14 to d 21	52.7	49.7	52.0	50.1	52.8	52.8	1.6	0.641	0.271	0.203	0.242	0.127
d 21 to d 27	73.9	64.9	65.0	67.3	72.6	66.8	4.6	0.618	0.182	0.191	0.460	0.404
d 7 to d 27	51.1	49.2	48.8	48.0	52.4	49.8	1.7	0.382	0.058	0.431	0.678	0.374
Overall	41.1	39.7	39.3	38.9	42.2	40.1	1.3	0.352	0.078	0.485	0.670	0.393
ADFI ^5^, g/d												
d 1 to d 7	16.1	15.3	16.5	16.6	16.3	16.0	0.8	0.533	0.541	0.557	0.284	0.398
d 7 to d 14	50.2	51.6	54.1	52.1	52.9	50.9	2.3	0.564	0.545	0.664	0.905	0.929
d 14 to d 21	80.9	80.2	78.8	80.7	82.1	81.0	2.5	0.547	0.606	0.848	0.706	0.653
d 21 to d 27	116.1	111.2	109.5	116.3	117.9	112.2	4.3	0.359	0.549	0.432	0.400	0.471
d 7 to d 27	81.9	82.6	80.2	80.1	85.3	81.3	3.1	0.434	0.319	0.871	0.919	0.855
Overall	61.8	62.2	60.6	60.7	64.5	61.5	2.4	0.405	0.344	0.907	0.998	0.788
G:F ^6^												
d 1 to d 7	0.757	0.770	0.720	0.759	0.749	0.731	0.030	0.889	0.796	0.762	0.506	0.424
d 7 to d 14	0.563	0.580	0.567	0.524	0.580	0.579	0.029	0.975	0.302	0.677	0.576	0.979
d 14 to d 21	0.654	0.619	0.660	0.621	0.646	0.658	0.023	0.964	0.540	0.305	0.407	0.261
d 21 to d 27	0.636	0.592	0.596	0.582	0.605	0.594	0.036	0.724	0.350	0.404	0.977	0.874
d 7 to d 27	0.625	0.599	0.609	0.606	0.614	0.613	0.019	0.947	0.548	0.355	0.607	0.566
Overall	0.666	0.641	0.648	0.649	0.655	0.652	0.020	0.959	0.573	0.393	0.647	0.620

^1^ Negative control, ^2^ Positive control (PC), ^3^ Bodyweight, ^4^ Average daily gain, ^5^ Average daily feed intake, ^6^ Gain to feed ratio.

**Table 3:** Alpha diversity of the mucosa-associated microbiota in the jejunum at the family and genus level of broiler chickens fed diets with phytase supplementation.

Item		Phytase, FTU/kg Feed			*p*-Value		
	NC ^1^	0 ^2^	2000	SEM	NC vs. 0	0 vs. 2000	NC vs. 2000
Family							
Chao1	60.50	50.32	67.51	5.46	0.182	0.040	0.371
Shannon	3.48	3.33	3.41	0.46	0.821	0.914	0.915
Simpson	0.78	0.75	0.81	0.09	0.846	0.640	0.776
Genus							
Chao1	68.16	50.57	70.16	7.62	0.109	0.091	0.855
Shannon	3.38	3.09	3.16	0.53	0.684	0.921	0.772
Simpson	0.73	0.70	0.77	0.11	0.802	0.641	0.819

^1^ Negative control, ^2^ Positive control (PC).

**Table 4:** Alpha diversity of the mucosa-associated microbiota in the jejunum at the family and genus level of broiler chickens fed diets with phytase supplementation.

Item		Phytase, FTU/kg Feed			*p*-Value		
	NC ^1^	0 ^2^	2000	SEM	NC vs. 0	0 vs. 2000	NC vs. 2000
Firmicutes	46.13	59.51	48.69	11.15	0.389	0.504	0.873
Proteobacteria	24.67	24.93	19.53	6.99	0.978	0.594	0.611
Cyanobacteria	16.47	8.67	15.91	6.78	0.409	0.778	0.290
Actinobacteria	12.03	6.40	10.34	3.25	0.221	0.407	0.719
Bacteroidetes	0.63	0.35	4.25	1.76	0.907	0.140	0.168
Others	0.84	0.16	0.52	0.35	0.178	0.491	0.528
Firmicutes to Bacteroidetes ratio	76.01	149.82	54.56	55.75	0.342	0.262	0.793

^1^ Negative control, ^2^ Positive control (PC).

**Table 5:** Relative abundance of the mucosa-associated microbiota in the jejunum at the family level of broiler chickens fed diets with phytase supplementation.

Item		Phytase, FTU/kg Feed			*p*-Value		
	NC ^1^	0 ^2^	2000	SEM	NC vs. 0	0 vs. 2000	NC vs. 2000
*Lactobacillaceae*	27.61	9.71	27.31	9.80	0.216	0.224	0.982
*Nostocaceae*	15.20	5.34	6.13	5.93	0.258	0.926	0.296
*Propionibacteriaceae*	6.58	8.10	1.85	2.34	0.651	0.078	0.173
*Comamonadaceae*	4.98	3.31	5.82	1.95	0.554	0.377	0.765
*Clostridiaceae*	4.93	11.29	4.92	2.67	0.113	0.112	0.996
*Moraxellaceae*	4.64	1.36	1.78	1.60	0.168	0.856	0.225
*Ruminococcaceae*	4.35	9.88	10.09	4.71	0.420	0.975	0.403
*Lachnospiraceae*	3.32	8.29	5.05	2.93	0.250	0.448	0.683
*Enterobacteriaceae*	2.38	1.86	1.40	0.54	0.580	0.844	0.095
*Microbacteriaceae*	2.20	1.06	1.47	0.68	0.257	0.684	0.457
*Rhodobacteraceae*	2.14	1.60	1.10	0.64	0.567	0.593	0.276
*Pseudomonadaceae*	1.47	0.53	1.15	0.61	0.298	0.487	0.719
*Sphingomonadaceae*	1.40	0.55	1.20	0.46	0.209	0.336	0.755
*Phormidiaceae*	1.24	1.32	0.71	0.45	0.902	0.359	0.424
*Helicobacteraceae*	0.05	8.93	0.11	3.01	0.054	0.055	0.986
*Prevotellaceae*	0.05	2.91	0.02	1.47	0.176	0.172	0.988
Others	17.43	23.83	29.81	9.54	0.642	0.680	0.385

^1^ Negative control, ^2^ Positive control (PC).

**Table 6:** Relative abundance of the mucosa-associated microbiota in the jejunum at the genus level of broiler chickens fed diets with phytase supplementation.

Item		Phytase, FTU/kg Feed			*p*-Value		
	NC ^1^	0 ^2^	2000	SEM	NC vs. 0	0 vs. 2000	NC vs. 2000
*Lactobacillus*	29.21	20.81	47.14	11.44	0.595	0.099	0.313
*Propionibacterium*	9.19	4.65	12.63	3.37	0.334	0.116	0.483
*Pelomonas*	6.52	10.01	2.64	2.38	0.296	0.046	0.268
*Clostridium*	4.86	10.38	8.43	3.35	0.242	0.688	0.464
*Faecalibacterium*	4.03	12.70	8.96	5.58	0.268	0.643	0.542
*Acinetobacter*	3.04	1.18	1.59	1.06	0.215	0.788	0.352
*Microbacterium*	2.73	3.03	0.66	0.88	0.809	0.080	0.121
*Pseudomonas*	2.32	2.02	0.31	0.78	0.782	0.143	0.090
*Methylobacterium*	1.42	1.54	0.27	0.44	0.843	0.065	0.092
*Sphingomonas*	1.14	1.03	0.19	0.34	0.811	0.107	0.071
*Enhydrobacter*	1.08	0.50	0.06	0.42	0.334	0.482	0.116
*Staphylococcus*	1.03	0.31	0.99	0.28	0.084	0.117	0.922
*Streptococcus*	0.87	1.08	0.47	0.53	0.786	0.441	0.605
*Corynebacterium*	0.87	0.42	0.45	0.24	0.201	0.921	0.258
*Stomatobaculum*	0.76	1.03	0.66	0.68	0.773	0.711	0.922
*Cupriavidus*	0.57	0.85	0.27	0.27	0.455	0.153	0.450
*Ruminococcus*	0.46	0.97	1.78	0.61	0.545	0.366	0.149
*Blautia*	0.30	0.87	1.33	0.47	0.382	0.500	0.143
*Butyricicoccus*	0.25	0.42	1.13	0.41	0.768	0.250	0.159
*Helicobacter*	0.19	10.67	0.88	4.19	0.085	0.121	0.908
*Prevotella*	0.16	0.05	3.46	1.53	0.957	0.138	0.151
*Mycoplasma*	0.01	0.29	0.14	0.18	0.282	0.565	0.640
Others	15.88	14.08	11.62	3.65	0.720	0.513	0.325

^1^ Negative control, ^2^ Positive control (PC).

**Table 7:** Relative abundance of the mucosa-associated microbiota in the jejunum at the species level of broiler chickens fed diets with phytase supplementation.

Item		Phytase, FTU/kg Feed			*p*-Value		
	NC ^1^	0 ^2^	2000	SEM	NC vs. 0	0 vs. 2000	NC vs. 2000
*Propionibacterium_acnes*	21.28	9.38	18.56	6.19	0.154	0.315	0.761
*Faecalibacterium_prausnitzii*	10.97	17.38	15.95	8.28	0.553	0.905	0.679
*Pelomonas_puraquae*	9.74	14.70	4.42	2.85	0.194	0.024	0.212
*Lactobacillus_vaginalis*	8.55	0.30	7.74	2.47	0.021	0.054	0.821
*Microbacterium_ginsengisoli*	4.25	3.87	1.00	1.01	0.766	0.068	0.041
*Lactobacillus_crispatus*	3.06	0.37	4.67	2.23	0.358	0.196	0.618
*Lactobacillus_reuteri*	2.90	0.11	3.09	1.17	0.083	0.097	0.914
*Pelomonas_aquatica*	2.78	2.63	1.31	0.65	0.864	0.176	0.137
*Enhydrobacter_aerosaccus*	2.50	1.21	0.17	1.06	0.354	0.500	0.144
*Acinetobacter_johnsonii*	1.47	0.22	2.15	0.72	0.199	0.084	0.521
*Arthrobacter_russicus*	0.86	0.79	0.10	0.32	0.864	0.158	0.122
*Clostridium_sp.*	0.75	2.23	1.76	1.09	0.305	0.766	0.526
*Cupriavidus_necator*	0.71	1.04	0.68	0.35	0.476	0.487	0.954
*Clostridium_spiroforme*	0.71	1.102	0.47	0.62	0.709	0.547	0.786
*Ruminococcus_torques*	0.66	0.64	7.99	3.03	0.995	0.112	0.113
*Clostridium_lactatifermentans*	0.58	1.30	0.16	0.79	0.491	0.333	0.716
*Butyricicoccus_pullicaecorum*	0.50	0.38	1.84	0.60	0.881	0.110	0.138
*Helicobacter_mastomyrinus*	0.49	8.94	0.14	3.68	0.094	0.117	0.947
*Enterococcus_cecorum*	0.37	0.95	0.71	0.42	0.303	0.692	0.589
*Clostridium_perfringens*	0.19	2.72	1.52	0.41	0.065	0.410	0.360
Others	26.7	29.83	29.56	8.62	0.777	0.982	0.817

^1^ Negative control, ^2^ Positive control (PC).

**Table 8:** Oxidative stress parameters of broiler chickens fed diets with phytase supplementation.

Item		Phytase, FTU/kg Feed			*p*-Value		
	NC ^1^	0 ^2^	2000	SEM	NC vs. 0	0 vs. 2000	NC vs. 2000
Malondialdehyde, µmol/g of protein	0.145	0.186	0.146	0.022	0.224	0.232	0.981
Protein carbonyl, nmol/mg of protein	2.222	2.497	2.410	0.148	0.210	0.685	0.385

^1^ Negative control, ^2^ Positive control (PC).

**Table 9:** Apparent ileal digestibility of DM, CP, Ca and P of broiler chickens fed diets with phytase supplementation.

Item		Phytase, FTU/kg Feed						*p*-Value				
	NC ^1^	0 ^2^	500	1000	2000	4000	SEM	Linear	Quadratic	NC vs. 0	0 vs. 1000 to 4000	0 vs. 2000 to 4000
DM ^3^, %	54.1	51.3	52.2	54.0	52.0	53.4	0.9	0.605	0.776	0.046	0.088	0.218
CP ^4^, %	67.8	65.4	69.7	70.6	69.5	70.0	1.4	0.129	0.187	0.244	0.008	0.018
Ca ^5^, %	51.4	45.9	50.1	50.1	50.6	52.3	3.5	0.745	0.832	0.262	0.204	0.196
P ^6^, %	42.1	36.6	39.6	40.0	40.1	43.7	2.3	0.941	0.607	0.092	0.086	0.070

^1^ Negative control, ^2^ Positive control (PC), ^3^ Dry matter, ^4^ Crude protein, ^5^ Calcium, ^6^ Phosphorus.

**Table 10:** Jejunal morphology of broiler chickens fed diets with phytase supplementation.

Item		Phytase, FTU/kg Feed						*p*-Value				
	NC ^1^	0 ^2^	500	1000	2000	4000	SEM	Linear	Quadratic	NC vs. 0	0 vs. 1000 to 4000	0 vs. 2000 to 4000
Villus height, μm	916	884	875	917	989	977	24	0.004	0.102	0.364	0.010	0.002
Villus width, μm	121	120	116	125	129	132	3	0.006	0.367	0.661	0.014	0.007
Crypt depth, μm	166	162	157	159	170	170	4	0.220	0.023	0.392	0.265	0.071
VH:CD ^3^	5.52	5.47	5.56	5.83	5.84	5.77	0.23	0.193	0.695	0.892	0.214	0.253

^1^ Negative control, ^2^ Positive control (PC), ^3^ Villus height to crypt depth ratio.

**Table 11:** Bone characteristics parameters of broiler chickens fed diets with phytase supplementation.

Item		Phytase, FTU/kg Feed						*p*-Value				
	NC ^1^	0 ^2^	500	1000	2000	4000	SEM	Linear	Quadratic	NC vs. 0	0 vs. 1000 to 4000	0 vs. 2000 to 4000
BBS ^3^, N	213.8	183.5	199.9	212.0	213.8	212.8	9.8	0.073	0.096	0.036	0.014	0.019
Tibia weight, g	4.0	4.3	4.4	4.1	4.0	4.3	0.2	0.444	0.445	0.404	0.476	0.505
Ash, %	35.6	34.2	35.1	35.5	35.3	36.0	0.6	0.670	0.942	0.093	0.039	0.045
Ca ^4^, % of ash	19.8	19.1	19.4	19.2	19.5	19.4	0.3	0.869	0.318	0.156	0.488	0.423
P ^5^, % of ash	10.1	9.5	10.0	9.9	10.0	9.9	0.1	0.646	0.417	0.012	0.036	0.034
Ash, g/tibia	8.4	8.2	8.9	8.8	8.5	9.8	0.3	0.703	0.111	0.763	0.015	0.007
Ca, g/tibia	0.83	0.77	1.01	0.75	0.79	0.78	0.05	0.355	0.667	0.615	0.996	0.912
P, g/tibia	0.42	0.40	0.52	0.39	0.41	0.40	0.04	0.356	0.562	0.699	0.950	0.959

^1^ Negative control, ^2^ Positive control (PC), ^3^ Bone breaking strength, ^4^ Calcium, ^5^ Phosphorus.

## Data Availability

The data presented in this study are available upon request from the corresponding author.
